# The impact of the environment and parental education on the emotional state of adolescents

**DOI:** 10.1192/j.eurpsy.2021.1904

**Published:** 2021-08-13

**Authors:** B. Kamberi

**Affiliations:** Psychiatry For Child And Adolescents, University Clinical Center of Kosovo, Pristina, Kosovo

**Keywords:** beli

## Abstract

**Introduction:**

Its basic object of study is the understanding of the individual and the group, Scientific direction in the study of behavior is an important feature of psychiatry.

**Objectives:**

Adolescence is a stage of physical and mental development of human, usually listed between the stage of childhood and legal maturity. in the middle aged 13 and 18 years the sexual way begins.

**Methods:**

Education is the primary category of pedagogical theory, which includes the concepts of education and teaching. Physical education is a subject taught in school to train students physically, to enable them to work and to defend themselves. The totality of spiritual, mental and physical qualities or qualities of family and society and learning and working.

**Results:**

By taking preventive measures to close educational institutions against the spread of the covid virus 19, it has affected the education system of students, teachers and parents because it has been physically disconnected from learning and has gone virtually ONLINE, where it has been a form of home isolation.

**Conclusions:**

Parenting is a constant concern for our entire society today, for child and adolescent psychiatry specialists and education experts, especially for all young parents.

**Disclosure:**

No significant relationships.

